# The role of the phonoaudiologist and the focus on ASD intervention

**DOI:** 10.1590/2317-1782/20212021264

**Published:** 2022-04-20

**Authors:** Fernanda Dreux Miranda Fernandes, Cibelle Albuquerque de la Higuera Amato, Jacy Perissinoto, Simone Aparecida Lopes-Herrera, Ana Paula Ramos de Souza, Ana Carina Tamanaha, Ana Cristina de Albuquerque Montenegro, Leticia Segeren, Fernanda Prada Machado, Bárbara Niegia Garcia de Goulart, Daniela Regina Molini-Avejonas

**Affiliations:** 1 Programa de Pós-graduação em Ciências da Reabilitação, Departamento de Fisioterapia, Fonoaudiologia e Terapia Ocupacional, Faculdade de Medicina, Universidade de São Paulo – USP - São Paulo (SP), Brasil.; 2 Programa de Pós-graduação em Distúrbios do Desenvolvimento, Centro de Ciências Biológicas e da Saúde, Universidade Presbiteriana Mackenzie – UPM - São Paulo (SP), Brasil.; 3 Programa de Pós-graduação em Distúrbios da Comunicação Humana, Departamento de Fonoaudiologia, Universidade Federal de São Paulo – UNIFESP - São Paulo (SP), Brasil.; 4 Programa de Pós-graduação em Fonoaudiologia, Departamento de Fonoaudiologia, Faculdade de Odontologia de Bauru, Universidade de São Paulo – USP - Bauru, SP, Brasil.; 5 Programa de Pós-graduação em Distúrbios da Comunicação Humana, Universidade Federal de Santa Maria – UFSM - Santa Maria, RS, Brasil.; 6 Departamento de Fonoaudiologia, Universidade Federal de Pernambuco – UFPE - Recife, PE, Brasil.; 7 Programa de Estudos Pós-graduados em Fonoaudiologia, Pontifícia Universidade Católica de São Paulo – PUC - São Paulo, SP, Brasil.; 8 Programa de Pós-graduação em Epidemiologia, Universidade Federal do Rio Grande do Sul – UFRGS - Porto Alegre, RS, Brasil.

The significant increase in the number of children diagnosed with Autism Spectrum Disorder (ASD) has generated many constructive discussions about the intervention alternatives offered to these individuals, unfortunately, there are also some controversial opinions on this matter. This letter to the editor is an initiative of a group of phonoaudiologist, researchers in this area, that strive to provide supporting elements for professional decision making in speech therapy.

There is much discussion regarding the symptomatology and characterization of ASD and, consequently, on priority areas for intervention. There have been consistent doubts expressed, and one of them concerns more directly the role played by phonoaudiologist in this context.

Language and communication difficulties are included in the criteria used to diagnose ASD, which Kanner^(1)^ initially named “Autistic Disturbances of Affective Contact”, and later “childhood autism”. These communication difficulties were emphasized by Kanner in his exquisite descriptions of the very first group of children he investigated: “there is little difference in communication skills between children with and without speech impairments”^(2)^.

For nearly eight decades, language and communication difficulties observed in ASD have been described with some variations and in different levels of specificity, but they leave no doubt as to the relevance of the role played by phonoaudiologist in the improvement of such condition descriptions, as well as in the assessment and treatment of language and communication disorders in ASD patients.

The Diagnostic and Statistical Manual of Mental Disorders (DSM) of the American Psychiatric Association (APA)^(3)^ and the International Classification of Diseases (ICD) of the World Health Organization (WHO)^(4)^ reflect the knowledge development about the subject. The last version of the DSM, published in 2013 (DMS-5)^(3)^ suggests that the criteria for ASD diagnosis should include two symptom clusters: ““persistent deficits in social communication”,” and ““restricted, repetitive patterns of behavior””.

The notion of “social communication” is not widely disseminated in actuality and it has been often stated that the DSM-5^(3)^ mentions “social and communication deficits”. This misinterpretation is further evidence of the relevance of the phonoaudiologist’ paticipation in multidisciplinary teams caring for this population. The American Speech-Language-Hearing Association (ASHA)^(5)^ offers a simple description: the concept of “social communication skills” includes skills such as variation in speech, perspective-taking, language comprehension and correct use of the verbal and nonverbal communication principles, as well as the proper use of the structural aspects of language (e.g., vocabulary, syntax, and phonology) to accomplish these goals. In language studies, this area is addressed by current pragmatic theories^(5)^.

Phonoaudiologists, as specialists in human communication^(6)^, are therefore the professionals capable of diagnosing, designing, proposing, and executing interventions related to the language and communication skills of individuals with ASD.

Another question that arises is: How should these attributions be inferred and dealt with? First and foremost, phonoaudiologist and other professionals who are part of multidisciplinary teams that care for individuals with ASD and their families must be well aware of the definitions of each element that comprise the Communication process. Although this is not an easy objective to achieve, it is necessary, because communication, speech and language have different key constituents that may impact the entire process of developing an intervention plan, while permeating all spheres of development and environments where these individuals and their families socialize. After all, we communicate all the time, and that is precisely the general purpose of an intervention process with ASD patients: the social use of language in an autonomous and personal way.

The phonoaudiologist uses the necessary scientific knowledge about language and communication^(6)^ to draw a detailed personal profile with skills and difficulties, that enables the development of a unique therapeutic plan according to the demands of each individual with ASD, taking their family and social context into consideration. The word “spectrum” evidences that ASD is manifested and developed differently in each case, providing each individual with characteristics that are common to the general condition, but also specific to each case..

Thus, it is clear that the use of a single therapeutic proposal - whether based on more or less structured methods, models or approaches - does not meet the particular needs demanded by the complexity of this clinical condition. In addition, it should be highlighted that the phonoaudiologist must be sensitive and attentive to all aspects of the individuality of a person with ASD^(7)^. There is no effective general approach for all children with ASD and their families, although there may be some convergence between them, focusing on the need for unique profiling of skills and difficulties^(8,9)^.

Unidirectional approaches limit the comprehensiveness of the care provided to each individual. The multidisciplinary perspective has great potential for innovative treatments and solutions, as it accommodates the specificity of each area, favoring the discussion in team meetings regarding action plans, consequently avoiding unilateral decisions^(10)^. In this scenario, the multidisciplinary team must analyze and choose, among the different therapeutic options, actions that best suit each individual with ASD and their family.

In this context, it is up to the phonoaudiologist to analyze the specificities of language and communication demanded by the individual with ASD at each moment of development. They are responsible for linking the diagnosis (personal profiling of skills and difficulties associated with language, communication and related aspects) to the therapeutic intervention proposal, based on clinical reasoning consistent with the scientific evidence that integratetheir respective clinical education. Interaction and respect between the professionals will contribute to a treatment that incorporates all areas of specialty.

Examples of different language and communication approaches developed by speech-language therapists with positive results can be found both in national and international literature^(11-23)^. There are studies addressing early detection and intervention^(11-13)^, family guidance^(14,15)^, functional approaches^(16)^, Augmentative and Alternative Communication (AAC)^(17-20)^, along with models and methods such as the ABA^(21)^, Denver^(22)^, DIR/Floortime^(23)^, PLAY Project^(24)^, among others, which show that phonoaudiologist play an important role in the elaboration of assessments and therapies specific to language, or even broader aspects in the development of individuals with ASD, and that they are able to choose the most adequate fit to each case amidst these numerous possibilities. Therefore, attempts to target a single therapeutic approach are not supported by scientific evidence; on the contrary, scientific evidence attributes to phonoaudiologist the ethical responsibility to make choices based on their broad and continuous education on the subject, thus assuming a critical and investigative approach, which constitutes the very essence of the scientific method.

In a national context where the main issue is the adequate provision of necessary services to all individuals with ASD and their families^(25)^, the respect for professional autonomy and practice based on scientific evidence should be the foundation for real efforts aimed at ensuring such rights.
