# Supplementary material for: The role of the phonoaudiologist and the focus on ASD intervention
Source: Codas. 2022 Apr 20;34(5):e20210264. doi: 10.1590/2317-1782/20212021264 (PMC9886177; doi:10.1590/2317-1782/20212021264)
Supplement: Translation

# Translation: O papel do fonoaudiólogo e o foco da intervenção no TEA

**Authors:** Fernanda Dreux Miranda Fernandes, Cibelle Albuquerque de la Higuera Amato, Jacy Perissinoto, Simone Aparecida Lopes-Herrera, Ana Paula Ramos de Souza, Ana Carina Tamanaha, Ana Cristina de Albuquerque Montenegro, Leticia Segeren, Fernanda Prada Machado, Bárbara Niegia Garcia de Goulart, Daniela Regina Molini-Avejonas

**Affiliations:** 1 Programa de Pós-graduação em Ciências da Reabilitação, Departamento de Fisioterapia, Fonoaudiologia e Terapia Ocupacional, Faculdade de Medicina, Universidade de São Paulo – USP - São Paulo (SP), Brasil.; 2 Programa de Pós-graduação em Distúrbios do Desenvolvimento, Centro de Ciências Biológicas e da Saúde, Universidade Presbiteriana Mackenzie – UPM - São Paulo (SP), Brasil.; 3 Programa de Pós-graduação em Distúrbios da Comunicação Humana, Departamento de Fonoaudiologia, Universidade Federal de São Paulo – UNIFESP - São Paulo (SP), Brasil.; 4 Programa de Pós-graduação em Fonoaudiologia, Departamento de Fonoaudiologia, Faculdade de Odontologia de Bauru, Universidade de São Paulo – USP - Bauru, SP, Brasil.; 5 Programa de Pós-graduação em Distúrbios da Comunicação Humana, Universidade Federal de Santa Maria – UFSM - Santa Maria, RS, Brasil.; 6 Departamento de Fonoaudiologia, Universidade Federal de Pernambuco – UFPE - Recife, PE, Brasil.; 7 Programa de Estudos Pós-graduados em Fonoaudiologia, Pontifícia Universidade Católica de São Paulo – PUC - São Paulo, SP, Brasil.; 8 Programa de Pós-graduação em Epidemiologia, Universidade Federal do Rio Grande do Sul – UFRGS - Porto Alegre, RS, Brasil.

O aumento significativo no número de crianças que recebem diagnósticos inseridos no espectro do autismo tem gerado discussões bem-vindas a respeito das alternativas de intervenção oferecidas a esse grupo e, infelizmente, também alguns posicionamentos questionáveis. Esta carta ao editor é uma iniciativa de um grupo de fonoaudiólogos, pesquisadores da área, que tem como objetivo trazer elementos para apoiar o fonoaudiólogo em sua tomada de decisões.

Há muitas discussões sobre a sintomatologia e caracterização do Transtorno do Espectro do Autismo e, consequentemente, sobre as áreas prioritárias de intervenção. Algumas dúvidas têm se repetido e uma delas diz respeito mais diretamente ao papel do fonoaudiólogo nesse contexto.

As dificuldades de linguagem e comunicação são parte dos critérios para o diagnóstico do que hoje é definido como Transtorno do Espectro do Autismo (TEA), denominado por Kanner^(1)^ de “Transtorno autista do contato afetivo” e depois de “autismo infantil”. Desde suas primorosas descrições do primeiro grupo de crianças, esse autor ressalta essas dificuldades de comunicação, mencionando que “existe pouca diferença no que diz respeito às habilidades de comunicação entre as crianças com e sem fala”^(2)^.

Durante as últimas quase oito décadas, as dificuldades de linguagem e comunicação observadas no TEA têm sido descritas com algumas variações e diferentes níveis de especificidade, mas não deixam dúvidas quanto à relevância do papel do fonoaudiólogo no avanço de tais descrições, na avaliação e terapia dos distúrbios de comunicação e linguagem de pessoas com TEA.

O Manual de Diagnóstico e Estatística dos Distúrbios Mentais (DSM), publicado pela Associação Americana de Psiquiatria (APA)^(3)^ e a Classificação Internacional de Doenças (CID), publicada pela Organização Mundial da Saúde (OMS)^(4)^, têm refletido a evolução no conhecimento a respeito do tema. A última versão do DSM^(3)^ sugere que os critérios para o diagnóstico de TEA incluem dois grupos de sintomas: “dificuldades persistentes em comunicação social” (*persistent deficits in social communication*) e “padrões de comportamento restritos e repetitivos” (*restricted, repetitive patterns of behavior*).

A noção de “*comunicação social*” não é amplamente difundida em nossa realidade e, muitas vezes, tem-se afirmado que o DSM5^(3)^ menciona “déficits sociais e de comunicação”. Essa falha de interpretação é mais uma evidência da relevância do fonoaudiólogo na equipe multidisciplinar de atenção a essa população. A Associação Americana de Fala, Linguagem e Audição^(5)^ (*ASHA – American Speech-Language and Hearing Associaton*) oferece uma descrição simples: o conceito de “habilidades de comunicação social” inclui a habilidade para variar o estilo de fala, considerar a perspectiva de outros, compreender e usar de forma apropriada as regras de comunicação verbal e não verbal e usar os aspectos estruturais da linguagem (por exemplo, vocabulário, sintaxe e fonologia) para atingir esses objetivos. Essa área, nos estudos de linguagem, é abordada pelas teorias pragmáticas^(5)^.

O fonoaudiólogo, como o especialista em comunicação humana^(6)^ é, assim, o profissional apto a diagnosticar, delinear, propor e executar a intervenção relacionada às habilidades de linguagem e comunicação das pessoas com TEA.

Temos aqui mais um ponto de dúvida: como definir e conduzir essas atribuições? Primeiramente, o fonoaudiólogo e demais profissionais que fazem parte das equipes que atuam com as pessoas com TEA, e com suas famílias, devem ter clareza quanto às definições de cada elemento que faz parte do processo de Comunicação. Não é um propósito fácil, mas é necessário, pois a comunicação, a fala e a linguagem apresentam constituintes diferenciais de base que podem impactar todo o processo de desenvolvimento de um planejamento de intervenção, ao mesmo tempo em que perpassam todas as esferas do desenvolvimento e todos os ambientes em que esse indivíduo e sua família convivem. Afinal, nos comunicamos o tempo todo e é exatamente esse o objetivo geral de um processo de intervenção com pessoas com TEA: o uso social da linguagem de forma autônoma e pessoal.

O fonoaudiólogo reúne o conhecimento científico necessário a respeito de comunicação e linguagem^(6)^ para traçar um perfil individual detalhado de habilidades e dificuldades que permite construir um planejamento terapêutico singular, de acordo com as demandas de cada indivíduo, considerando seu contexto familiar e social. A palavra “espectro” evidencia que o TEA se manifesta e se desenvolve de formas diferentes em cada caso, proporcionando que cada pessoa tenha características em comum com o quadro geral, mas também individuais.

Deste modo, fica claro que o emprego de uma única proposta terapêutica – seja ela baseada em métodos, modelos ou abordagens mais ou menos estruturados - não responde às necessidades particulares determinadas pela complexidade do quadro clínico de TEA. Além disso, é importante destacar que o terapeuta deve ser sensível e estar atento a todos os aspectos da individualidade da pessoa com TEA^(7)^. Não há uma abordagem que seja efetiva para todas as crianças e suas famílias, embora possam existir convergências entre elas, com foco no perfil singular de habilidades e dificuldades^(8,9)^.

Abordagens unidirecionais limitam a integralidade do cuidado do indivíduo. A perspectiva multiprofissional propicia um grande potencial de tratamentos e soluções inovadoras, pois pode manter a especificidade de cada área, favorecendo a discussão das ações em equipe, evitando-se decisões unilaterais^(10)^. É neste cenário que a equipe multidisciplinar precisa analisar, dentre as diferentes opções terapêuticas, as que mais se adequem a cada pessoa com TEA e sua família.

Nesse contexto, cabe ao fonoaudiólogo analisar as especificidades da comunicação e da linguagem demandadas pelo indivíduo a cada momento do desenvolvimento. É dele a responsabilidade de articular o diagnóstico (perfil individualizado de habilidades e dificuldades relacionadas à comunicação e linguagem e aspectos correlatos) e a proposta de intervenção terapêutica, a partir de raciocínio clínico coerente com as evidências científicas que compõem sua formação clínica específica. A interação e o respeito entre todos os profissionais contribuirão para um tratamento que integre as especialidades.

A literatura nacional e internacional^(11-23)^ apresenta exemplos de diferentes abordagens de comunicação e linguagem, desenvolvidas por fonoaudiólogos, com resultados positivos. Há pesquisas em detecção e intervenção precoce^(11-13)^, orientação familiar^(14,15)^, abordagem funcional^(16)^, Comunicação Suplementar Alternativa (CSA)^(17-20)^, e modelos, métodos e abordagens como ABA^(21)^, Denver^(22)^, DIR-Floortime^(23)^, Play project^(24)^, entre outros, que evidenciam que o fonoaudiólogo tem participação importante na construção de avaliações e terapêuticas específicas à linguagem ou mesmo mais amplas do desenvolvimento de indivíduos com TEA, sendo capaz de escolher a mais pertinente a cada caso, a partir dessas inúmeras possibilidades. Deste modo, tentativas de direcionamento para uma única abordagem terapêutica, não se sustentam em evidências científicas. Ao contrário, as evidências científicas atribuem ao fonoaudiólogo o compromisso ético de fazer escolhas a partir de formação ampla e continuada no tema, assumindo um caráter crítico e investigativo, o que constitui a essência da postura científica.

Num contexto nacional em que a grande questão é a oferta adequada dos serviços necessários a todas as pessoas com TEA e suas famílias^(25)^, o respeito à autonomia profissional e a prática baseada em evidências científicas deveriam ser a base dos verdadeiros esforços dirigidos à garantia desses direitos.
